# Supplementary material for: Emotional Imagery Influences the Adaptive Force in Young Women: Unpleasant Imagery Reduces Instantaneously the Muscular Holding Capacity
Source: Brain Sci. 2022 Sep 29;12(10):1318. doi: 10.3390/brainsci12101318 (PMC9599475; doi:10.3390/brainsci12101318)
Supplement: Supplementary file 1 [file brainsci-12-01318-s001.zip › brainsci-1913610-supplementary.pdf]

brain sciences

Supplementary Material

Article

Emotional imagery influences the Adaptive Force in young women: unpleasant imagery reduces instantaneously the muscular holding capacity

Schaefer LV, Dech S, Wolff LL, Bittmann FN

Content

Table S1. Anthropometric data 2

Table S2. Tester's ratings of manual muscle tests 3

Table S3. Maximal Adaptive Force (AF_{max}) of elbow flexors 4

Table S4. Maximal Adaptive Force (AF_{max}) of hip flexors 5

Table S5. Maximal isometric Adaptive Force ($AFiso_{max}$) of elbow flexors 6

Table S6. Maximal isometric Adaptive Force ($AFiso_{max}$) of hip flexors 7

Table S7. Adaptive Force at onset of oscillations (AFosc) of elbow flexors 8

Table S8. Adaptive Force at onset of oscillations (AFosc) of hip flexors 9

Table S9. Slope of force rise of elbow flexors 10

Table S10. Slope of force rise of hip flexors 11

Table S1. Anthropometric data

Table S1. Anthropometric data of each participant: gender (female = 1), age (years), height (cm), mass (kg)) including arithmetic mean (M) and standard deviation (SD).

participant	gender	age (yrs.)	height (cm)	mass (kg)
1	1	22	180	80
2	1	25	165	58
3	1	23	171	63
4	1	30	177	72
5	1	26	180	70
6	1	26	160	54
7	1	20	177	69
8	1	25	168	60
9	1	23	177	68
10	1	20	170	60
11	1	30	165	57
12	1	29	158	58
M		24.92	170.67	64.08
SD		3.50	7.63	7.69

Table S2. Tester's ratings of manual muscle tests

Table S2. Results of tester's manual muscle test (MMT) ratings of elbow (1) and hip (2) flexors: unstable = 0, stable = 1, indifferent = 2 of baseline (base), pleasant (pleas) and unpleasant (unpl) imagery for each trial (_1 to _3 refers to number of trial) of each participant.

Partici-pant	tested muscle	base_1	base_2	pleas_1	pleas_2	pleas_3	unpl_1	unpl_2	unpl_3
**elbow flexors**									
1	1	1	1	1	1	0	1	1	0
2	1	1	1	1	1	1	0	0	0
3	1	1	1	1	0	1	0	0	0
4	1	1	1	1	1	1	0	0	0
5	1	1	1	1	1	1	0	0	0
6	1	1	1	1	1	1	0	0	0
7	-	-	-	-	-	-	-	-	-
8	1	1	1	1	1	1	0	0	0
9	1	1	-	1	1	0	0	0	0
10	-	-	-	-	-	-	-	-	-
11	1	1	1	-	1	1	0	0	1
12	1	1	1	1	1	1	0	0	0
**hip flexors**									
1	2	-	-	-	-	-	-	-	-
2	2	1	1	1	1	1	0	0	0
3	2	1	1	1	1	1	0	-	0
4	2	1	1	1	1	1	0	0	0
5	2	1	1	1	-	1	0	0	0
6	2	1	1	1	1	0	0	2	0
7	2	-	1	1	1	1	0	0	1
8	2	1	1	1	-	1	0	0	0
9	2	1	1	1	1	0	0	1	0
10	2	1	1	1	-	0	0	2	0
11	2	1	1	1	1	1	0	0	0
12	2	1	1	0	1	1	0	2	0

Table S3. *Maximal Adaptive Force (AF_{max}) (N) of elbow flexors for baseline as well as pleasant and unpleasant imagery for each participant and trial. The maximal value of all trials ($maxAF_{max}$) is given, too. Furthermore, the individual and group arithmetic means (M), standard deviations (SD) and coefficient of variation (CV) are displayed.*

elbow flexors

	**baseline**		**pleasant**			**unpleasant**			**baseline**			**pleasant**			**unpleasant**			**$maxAF_{max}$**
	**M1**	**M2**	**M1**	**M2**	**M3**	**M1**	**M2**	**M3**	**M**	**SD**	**CV**	**M**	**SD**	**CV**	**M**	**SD**	**CV**	
1	134.28	133.33	176.60	138.36	256.91	166.30	149.94	242.82	133.80	0.68	0.01	190.62	60.50	0.32	186.35	49.58	0.27	256.91
2	180.98	216.94	182.07	201.48	217.72	137.94	142.98	136.42	198.96	25.43	0.13	200.42	17.85	0.09	139.11	3.44	0.02	217.72
3	152.78	153.43	142.58	157.58	151.04	156.46	143.35	138.18	153.10	0.46	0.00	150.40	7.52	0.05	146.00	9.42	0.06	157.58
4	159.87	179.68	219.803	192.634	184.99	175.09	243.62	202.40	169.78	14.01	0.08	199.14	18.30	0.09	207.03	34.50	0.17	243.62
5	161.99	148.69	133.893	151.39	167.05	187.33	189.03	181.66	155.34	9.41	0.06	150.78	16.59	0.11	186.01	3.86	0.02	189.03
6	155.31	133.08	148.50	143.79	157.88	206.32	185.43	177.10	144.20	15.72	0.11	150.05	7.17	0.05	189.62	15.05	0.08	206.32
7	-	-	-	-	-	-	-	-	-	-	-	-	-	-	-	-	-	-
8	78.36	91.76	92.75	93.90	101.61	140.11	127.82	162.36	85.06	9.47	0.11	96.09	4.82	0.05	143.43	17.51	0.12	162.36
9	164.91	-	175.94	172.37	185.04	194.60	184.76	179.21	164.91	-	-	177.79	6.53	0.04	186.19	7.79	0.04	194.60
10	-	-	-	-	-	-	-	-	-	-	-	-	-	-	-	-	-	-
11	166.57	200.72	-	154.31	142.89	158.48	132.85	101.84	183.64	24.15	0.13	148.60	8.08	0.05	131.06	28.36	0.22	200.72
12	187.59	172.32	168.58	166.52	167.86	168.25	169.76	195.47	179.96	10.80	0.06	167.65	1.04	0.01	177.83	15.30	0.09	195.47
								M	156.87			163.15			169.26			202.43
								SD	31.83			31.39			26.56			31.31
								CV	0.20			0.19			0.16			0.15

Table S4. *Maximal Adaptive Force (AF_{max}) (N) of hip flexors for baseline as well as pleasant and unpleasant imagery for each participant and trial. The maximal value of all trials ($maxAF_{max}$) is given, too. Furthermore, the individual and group arithmetic means (M), standard deviations (SD) and coefficient of variation (CV) are displayed.*

	**hip flexors**																		
	**baseline**		**pleasant**			**unpleasant**			**baseline**			**pleasant**			**unpleasant**			**$maxAF_{max}$**
	**M1**	**M2**	**M1**	**M2**	**M3**	**M1**	**M2**	**M3**	**M**	**SD**	**CV**	**M**	**SD**	**CV**	**M**	**SD**	**CV**	
1	-	-	-	-	-	-	-	-	-	-	-	-	-	-	-	-	-	-
2	200.02	171.11	192.09	208.92	174.84	160.75	186.32	173.03	185.56	20.44	0.11	191.95	17.04	0.09	173.37	12.79	0.07	208.92
3	184.54	155.64	169.87	189.10	190.14	191.20	-	166.16	170.09	20.43	0.12	183.03	11.42	0.06	178.68	17.71	0.10	191.20
4	149.36	172.58	217.09	205.66	186.63	243.10	229.11	251.44	160.97	16.42	0.10	203.13	15.39	0.08	241.22	11.28	0.05	251.44
5	167.05	156.08	166.10	-	143.41	181.07	215.89	225.55	161.57	7.76	0.05	154.76	16.05	0.10	207.50	23.40	0.11	225.55
6	168.04	121.55	113.60	127.77	205.79	143.60	103.26	205.84	144.80	32.88	0.23	149.05	49.64	0.33	150.90	51.68	0.34	205.84
7	-	161.92	159.94	180.48	160.05	226.40	215.95	178.12	161.92	-	-	166.82	11.83	0.07	206.82	25.40	0.12	226.40
8	132.87	100.45	93.83	-	89.96	192.85	185.90	203.19	116.66	22.93	0.20	91.90	2.74	0.03	193.98	8.70	0.04	203.19
9	155.62	150.77	147.84	171.14	234.10	187.86	133.33	200.37	153.20	3.43	0.02	184.36	44.63	0.24	173.85	35.64	0.21	234.10
10	178.00	168.49	196.12	-	249.70	259.60	181.20	207.92	173.25	6.72	0.04	222.91	37.89	0.17	216.24	39.85	0.18	259.60
11	144.25	152.97	153.11	143.64	134.19	175.93	156.58	147.80	148.61	6.17	0.04	143.65	9.46	0.07	160.10	14.39	0.09	175.93
12	164.07	169.85	202.91	134.71	152.08	147.43	147.09	169.04	166.96	4.08	0.02	163.23	35.44	0.22	154.52	12.58	0.08	202.91
								M	158.51			168.62			187.02			216.82
								SD	18.02			35.01			28.58			25.27
								CV	0.11			0.21			0.15			0.12

Table S5. *Maximal isometric Adaptive Force ($AFiso_{max}$) (N) of elbow flexors for baseline as well as pleasant and unpleasant imagery for each participant and trial. The individual and group arithmetic means (M), standard deviations (SD) and coefficient of variation (CV) are given.*

	**baseline**		**pleasant**			**unpleasant**			**baseline**			**pleasant**			**unpleasant**		
	**M1**	**M2**	**M1**	**M2**	**M3**	**M1**	**M2**	**M3**	**M**	**SD**	**CV**	**M**	**SD**	**CV**	**M**	**SD**	**CV**
1	134.28	133.33	176.60	138.36	156.01	166.30	149.94	119.93	133.80	0.68	0.01	156.99	19.14	0.12	145.39	23.52	0.16
2	180.98	216.94	182.07	201.48	217.72	75.68	66.98	77.52	198.96	25.43	0.13	200.42	17.85	0.09	73.40	5.63	0.08
3	152.78	153.43	142.58	108.16	151.04	98.85	125.05	100.20	153.10	0.46	0.00	133.92	22.71	0.17	108.03	14.75	0.14
4	159.87	179.68	219.80	192.63	184.99	42.64	116.40	40.43	169.78	14.01	0.08	199.14	18.30	0.09	66.49	43.24	0.65
5	161.99	148.69	133.89	151.39	167.05	54.91	39.78	62.72	155.34	9.41	0.06	150.78	16.59	0.11	52.47	11.66	0.22
6	155.31	133.08	148.50	143.79	157.88	139.75	116.17	87.29	144.20	15.72	0.11	150.05	7.17	0.05	114.40	26.28	0.23
7	-	-	-	-	-	-	-	-	-	-	-	-	-	-	-	-	-
8	78.36	91.76	92.75	93.90	101.61	32.22	33.08	33.88	85.06	9.47	0.11	96.09	4.82	0.05	33.06	0.83	0.03
9	164.91	-	175.94	172.37	156.75	114.63	134.60	113.35	164.91	-	-	168.36	10.21	0.06	120.86	11.92	0.10
10	-	-	-	-	-	-	-	-	-	-	-	-	-	-	-	-	-
11	166.57	200.72	-	154.31	142.89	81.00	118.42	101.84	183.64	24.15	0.13	148.60	8.08	0.05	100.42	18.75	0.19
12	187.59	172.32	168.58	166.52	167.86	79.33	94.81	95.68	179.96	10.80	0.06	167.65	1.04	0.01	89.94	9.20	0.10
								M	156.87			157.20			90.45		
								SD	31.83			30.38			34.20		
								CV	0.20			0.19			0.38		

Table S6. *Maximal isometric Adaptive Force ($AFiso_{max}$) (N) of hip flexors for baseline as well as pleasant and unpleasant imagery for each participant and trial. The individual and group arithmetic means (M), standard deviations (SD) and coefficient of variation (CV) are given.*

	**baseline**		**pleasant**			**unpleasant**			**baseline**			**pleasant**			**unpleasant**		
	**M1**	**M2**	**M1**	**M2**	**M3**	**M1**	**M2**	**M3**	**M**	**SD**	**CV**	**M**	**SD**	**CV**	**M**	**SD**	**CV**
1	-	-	-	-	-	-	-	-	-	-	-	-	-	-	-	-	-
2	200.02	171.11	192.09	208.92	174.84	55.37	83.08	104.99	185.56	20.44	0.11	191.95	17.04	0.09	81.14	24.87	0.31
3	184.54	155.64	169.87	189.10	190.14	156.84	-	102.97	170.09	20.43	0.12	183.03	11.42	0.06	129.90	38.09	0.29
4	149.36	147.75	217.09	205.66	186.63	54.77	81.34	80.03	148.55	1.14	0.01	203.13	15.39	0.08	72.05	14.98	0.21
5	167.05	156.08	166.10	-	143.41	118.42	113.06	100.81	161.57	7.76	0.05	154.76	16.05	0.10	110.76	9.03	0.08
6	168.04	121.55	113.60	127.77	128.66	64.30	103.26	116.85	144.80	32.88	0.23	123.34	8.45	0.07	94.80	27.28	0.29
7		161.92	159.94	180.48	160.05	210.10	173.64	178.12	161.92	-	-	166.82	11.83	0.07	187.28	19.88	0.11
8	132.87	100.45	93.83		89.96	31.48	30.53	34.84	116.66	22.93	0.20	91.90	2.74	0.03	32.28	2.27	0.07
9	155.62	150.77	147.84	171.14	135.36	110.01	133.33	121.98	153.20	3.43	0.02	151.45	18.16	0.12	121.77	11.67	0.10
10	178.00	168.49	196.12		179.57	207.01	181.20	160.91	173.25	6.72	0.04	187.84	11.70	0.06	183.04	23.10	0.13
11	144.25	152.97	153.11	143.64	134.19	136.82	110.39	103.95	148.61	6.17	0.04	143.65	9.46	0.07	117.05	17.42	0.15
12	164.07	169.85	130.04	134.71	152.08	105.95	132.89	139.45	166.96	4.08	0.02	138.94	11.61	0.08	126.10	17.75	0.14
								M	157.38			157.89			114.20		
								SD	18.24			33.13			45.28		
								CV	0.12			0.21			0.40		

Table S7. *Adaptive Force at onset of oscillations (AFosc) (N) of elbow flexors for baseline as well as pleasant and unpleasant imagery for each participant and trial. The individual and group arithmetic means (M), standard deviations (SD) and coefficient of variation (CV) are given.*

	**baseline**		**pleasant**			**unpleasant**			**baseline**			**pleasant**			**unpleasant**		
	**M1**	**M2**	**M1**	**M2**	**M3**	**M1**	**M2**	**M3**	**M**	**SD**	**CV**	**M**	**SD**	**CV**	**M**	**SD**	**CV**
1	96.79	107.32	169.64	114.75	256.91	158.59	149.94	242.82	102.05	7.44	0.07	180.43	71.69	0.40	183.78	51.31	0.28
2	113.80	216.94	157.05	157.16	132.31	122.76	140.19	127.07	165.37	72.93	0.44	148.84	14.32	0.10	130.01	9.08	0.07
3	140.62	151.20	130.08	133.17	130.82	152.34	139.26	138.18	145.91	7.48	0.05	131.35	1.61	0.01	143.26	7.88	0.06
4	117.37	171.28	203.39	167.27	158.65	143.66	235.41	170.34	144.33	38.12	0.26	176.44	23.74	0.13	183.13	47.20	0.26
5	90.06	122.53	110.97	131.83	118.54	187.33	189.03	181.66	106.29	22.96	0.22	120.45	10.56	0.09	186.01	3.86	0.02
6	132.82	81.93	102.54	130.46	127.98	206.32	185.43	177.10	107.38	35.99	0.34	120.32	15.45	0.13	189.62	15.05	0.08
7	-	-	-	-	-	-	-	-	-	-	-	-	-	-	-	-	-
8	57.25	56.37	65.83	63.47	53.60	130.88	122.18	161.15	56.81	0.63	0.01	60.97	6.49	0.11	138.07	20.46	0.15
9	100.65	-	139.75	96.48	180.39	181.22	176.51	179.21	100.65	-	-	138.87	41.97	0.30	178.98	2.36	0.01
10	-	-	-	-	-	-	-	-	-	-	-	-	-	-	-	-	-
11	142.36	96.97	-	116.52	89.26	140.05	111.43	90.89	119.67	32.09	0.27	102.89	19.27	0.19	114.12	24.69	0.22
12	113.86	107.85	125.79	148.34	133.36	162.08	147.87	195.47	110.85	4.25	0.04	135.83	11.47	0.08	168.47	24.43	0.15
								M	115.93			131.64			161.54		
								SD	30.33			34.69			27.54		
								CV	0.26			0.26			0.17		

Table S8. *Adaptive Force at onset of oscillations (AFosc) (N) of hip flexors for baseline as well as pleasant and unpleasant imagery for each participant and trial. The individual and group arithmetic means (M), standard deviations (SD) and coefficient of variation (CV) are given.*

	**baseline**		**pleasant**			**unpleasant**			**baseline**			**pleasant**			**unpleasant**		
	**M1**	**M2**	**M1**	**M2**	**M3**	**M1**	**M2**	**M3**	**M**	**SD**	**CV**	**M**	**SD**	**CV**	**M**	**SD**	**CV**
1	-	-	-	-	-	-	-	-	-	-	-	-	-	-	-	-	-
2	146.34	130.52	179.23	109.27	124.42	160.75	184.39	156.40	138.43	11.18	0.08	137.64	36.80	0.27	167.18	15.06	0.09
3	148.75	147.11	166.77	174.28	190.14	131.95	-	153.28	147.93	1.16	0.01	177.06	11.93	0.07	142.61	15.08	0.11
4	92.19	128.50	120.94	194.83	123.58	159.36	222.24	98.22	110.35	25.67	0.23	146.45	41.92	0.29	159.94	62.01	0.39
5	125.92	81.56	154.07	-	71.94	74.79	178.95	96.84	103.74	31.37	0.30	113.00	58.08	0.51	116.86	54.89	0.47
6	109.37	94.87	78.83	77.87	205.84	127.65	60.07	197.43	102.12	10.25	0.10	120.85	73.61	0.61	128.38	68.69	0.54
7		127.19	89.32	132.08	134.50	193.36	188.78	116.63	127.19	-	-	118.63	25.41	0.21	166.26	43.04	0.26
8	67.15	48.89	45.39		47.06	178.68	185.90	134.98	58.02	12.91	0.22	46.22	1.18	0.03	166.52	27.55	0.17
9	67.57	64.48	70.02	78.37	142.22	91.56	104.19	114.18	66.02	2.18	0.03	96.87	39.49	0.41	103.31	11.34	0.11
10	130.99	106.63	121.19		108.13	115.95	133.42	195.57	118.81	17.23	0.14	114.66	9.24	0.08	148.31	41.85	0.28
11	82.02	75.35	98.27	100.10	108.22	95.04	104.92	96.40	78.69	4.71	0.06	102.20	5.30	0.05	98.79	5.36	0.05
12	121.04	97.57	189.06	116.44	147.25	96.40	129.30	133.53	109.31	16.60	0.15	150.91	36.45	0.24	119.74	20.33	0.17
								M	105.51			120.41			137.99		
								SD	28.49			34.05			25.86		
								CV	0.27			0.28			0.19		

Table S9. *Logarithmic slope of force rise (lg(N/s)) of elbow flexors for baseline as well as pleasant and unpleasant imagery for each participant and trial. The individual and group arithmetic means (M), standard deviations (SD) and coefficient of variation (CV) are given.*

	**baseline**		**pleasant**			**unpleasant**			**baseline**			**pleasant**			**unpleasant**		
	**M1**	**M2**	**M1**	**M2**	**M3**	**M1**	**M2**	**M3**	**M**	**SD**	**CV**	**M**	**SD**	**CV**	**M**	**SD**	**CV**
1	1.78	1.90	2.07	1.96	2.00	2.08	2.09	2.11	1.84	0.09	0.05	2.01	0.06	0.03	2.09	0.02	0.01
2	1.68	2.00	1.95	2.01	1.99	1.87	1.89	1.97	1.84	0.22	0.12	1.98	0.03	0.02	1.91	0.05	0.03
3	2.11	1.87	1.94	1.93	1.89	1.97	2.02	1.78	1.99	0.17	0.09	1.92	0.02	0.01	1.92	0.13	0.07
4	2.01	1.98	1.95	1.93	2.06	1.95	1.94	1.90	2.00	0.02	0.01	1.98	0.07	0.04	1.93	0.03	0.01
5	1.74	1.79	1.78	1.92	1.76	1.82	1.79	1.76	1.77	0.04	0.02	1.82	0.09	0.05	1.79	0.03	0.02
6	1.98	-	1.88	1.88	2.01	2.06	1.82	1.92	1.98	-	-	1.92	0.08	0.04	1.93	0.12	0.06
7	-	-	-	-	-	-	-	-	-	-	-	-	-	-	-	-	-
8	1.55	1.65	1.49	1.53	1.42	1.37	1.50	1.43	1.60	0.07	0.04	1.48	0.06	0.04	1.43	0.06	0.04
9	1.71	-	1.79	-	1.94	1.90	1.85	1.85	1.71	-	-	1.86	0.10	0.06	1.87	0.03	0.02
10	-	-	-	-	-	-	-	-	-	-	-	-	-	-	-	-	-
11	1.73	1.98	-	1.88	1.91	1.96	1.98	-	1.85	0.18	0.09	1.89	0.02	0.01	1.97	0.01	0.01
12	1.86	1.90	1.93	2.06	2.00	1.85	1.92	1.85	1.88	0.03	0.02	2.00	0.07	0.03	1.87	0.04	0.02
								M	1.85			1.89			1.87		
								SD	0.13			0.16			0.17		
								CV	0.07			0.08			0.09		

Table S10. *Logarithmic slope of force rise (lg(N/s)) of hip flexors for baseline as well as pleasant and unpleasant imagery for each participant and trial. The individual and group arithmetic means (M), standard deviations (SD) and coefficient of variation (CV) are given.*

	**baseline**		**pleasant**			**unpleasant**			**baseline**			**pleasant**			**unpleasant**		
	**M1**	**M2**	**M1**	**M2**	**M3**	**M1**	**M2**	**M3**	**M**	**SD**	**CV**	**M**	**SD**	**CV**	**M**	**SD**	**CV**
1	-	-	-	-	-	-	-	-	-	-	-	-	-	-	-	-	-
2	2.06	1.85	1.86	1.99	2.00	1.68	2.15	1.98	1.96	0.15	0.08	1.95	0.08	0.04	1.94	0.24	0.12
3	2.02	1.96	1.99	2.13	1.99	2.02	-	1.92	1.99	0.04	0.02	2.04	0.08	0.04	1.97	0.07	0.04
4	2.07	1.91	2.14	2.12	1.87	1.74	1.92	1.95	1.99	0.11	0.06	2.05	0.15	0.07	1.87	0.12	0.06
5	1.86	1.61	1.95	-	-	1.85	2.00	-	1.73	0.18	0.10	1.95	-	-	1.93	0.10	0.05
6	1.94	2.12	-	-	1.78	2.12	-	1.96	2.03	-	-	1.78	-	-	2.04	0.11	0.06
7	-	-	-	-	-	1.97	1.96	-	-	-	-	-	-	-	1.97	0.01	0.01
8	1.60	1.54	1.46	-	1.47	1.54	1.46	1.44	1.57	0.05	0.03	1.46	0.01	0.01	1.48	0.05	0.04
9	-	-	1.72	-	2.01	1.90	-	1.90	-	-	-	1.87	0.21	0.11	1.90	0.00	0.00
10	-	-	-	-	1.94	2.03	-	1.88	-	-	-	1.94	-	-	1.96	0.10	0.05
11	1.88	-	1.85	1.91	1.92	1.99	1.92	-	1.88	-	-	1.89	0.03	0.02	1.95	0.05	0.03
12	1.89	1.84	1.96	-	2.04	-	1.92	1.99	1.86	0.04	0.02	2.00	0.06	0.03	1.95	0.05	0.03
								M	1.88			1.89			1.90		
								SD	0.16			0.17			0.15		
								CV	0.08			0.09			0.08		
